# Incidental detection of multiple endocrine neoplasia and medullary thyroid carcinoma before starting GLP-1 agonist: A case report

**DOI:** 10.1016/j.heliyon.2024.e33420

**Published:** 2024-06-22

**Authors:** Katie Glasgow, Victoria Jiminez, Natalie Garcia, Andrea Gillis

**Affiliations:** a Department of Surgery, University of Utah, USA; b Heersink School of Medicine, University of Alabama at Birmingham, USA; c Department of Dermatology, Baylor College of Medicine, USA; d Department of Surgery, University of Alabama at Birmingham, USA

**Keywords:** Medullary thyroid carcinoma, GLP-1 agonist, Multiple endocrine neoplasia

## Abstract

A man, in his 30s, with a history of obesity and hypothyroidism planned to begin taking a new Glucagon-like peptide-1 (GLP-1) agonist for weight loss. As these medications have been associated with C-cell hyperplasia, a calcitonin level was checked as evaluation prior to starting the drug. This returned at 131 pg/mL (upper limit of normal<10 pg/mL), and a subsequent carcinoembryonic antigen was 5.2 ng/mL (ref<3 ng/mL). Thyroid ultrasound was performed and demonstrated bilateral subcentimeter nodules. After total thyroidectomy, final pathology demonstrated bilateral 0.8 cm medullary thyroid carcinoma. Genetic testing revealed a NM_020975.6: c.1826G > A; p.Cys609Tyr. germline RET mutation, confirming the diagnosis of multiple endocrine neoplasia 2 syndrome. The patient recovered well from treatment. His first-degree relatives also underwent genetic testing. This case represents a surprising diagnosis of familial multiple endocrine neoplasia 2A prior to starting a Glucagon-like peptide-1 agonist.

## 1 Introduction

Multiple endocrine neoplasia (MEN) are rare autosomal dominant genetic syndromes characterized by occurrence of specific types of endocrine tumors or proliferation disorders. MEN syndromes are now comprised of four subtypes: MEN 1, which results from a mutation in the MENIN gene, MEN 2 (formerly 2A) and MEN 3 (formerly 2B) resulting from mutations in the *RET* proto-oncogene, and MEN 4 resulting from mutations in the *CDKN1B* gene [1,2]. MEN 2 is specifically characterized by the co-occurrence of pheochromocytoma, medullary thyroid cancer (MTC), and parathyroid hyperplasia [3]. The North American Association of Central Cancer Registries estimated there to be 0.24 cases per 100,000 people of medullary thyroid cancer in 2020 [4]. Because *RET* genes normally regulate maturation in neural crest cells, mutations effect tissues derived from the neural crest origin, including thyroid parafollicular “C” cells [5]. These cells normally produce small amounts of calcitonin, but produce larger amounts in MTC. Therefore, increases in calcitonin may be a confirmatory marker of disease before macroscopic nodules develop [2,6]. Genotypic variations in codon-specific *RET* mutations have been found to correlate directly to varying risk of development of MEN 2 and MEN 3 phenotypes.

MEN 2 syndrome has very high penetrance and variable expressivity. It affects 60 %–90 % of families with *RET* mutations, and medullary thyroid cancer (MTC) is the most common, and typically first, manifestation of MEN 2 [7]. MTC is an aggressive form of thyroid cancer that drives the need for early diagnosis and screening, as the only curative treatment is surgery. Many patients with MTC present late in the disease course with compressive symptoms due to mass effect because systemic symptoms such as fatigue, weight loss, and bone pain are often unreported or attributed to more commonplace illnesses. Up to 70 % of patients with MTC who present with a palpable thyroid nodule have cervical metastases and 10 % have distant metastases [4]. The treatment for diagnosed MTC is total thyroidectomy with central neck dissection and lateral neck dissection if calcitonin is over 500 or other clinical exam or imaging findings raise concern for MTC spread [4]. MTC can be prevented by early total thyroidectomy by age 5 for MEN 2 and age 1 for MEN 3 [4]. Typical screening for MTC recurrence in MEN patients includes monitoring lab values of calcitonin and yearly neck ultrasound, if prophylactic thyroidectomy is not performed [4].

There are few reported cases of calcitonin negative MTC, which can complicate the diagnosis, prognostication, and follow up of these patients [8]. Most of these patients did show similar histological patterns and a positive immunostaining for either chromogranin A, somatostatin and neuron specific enolase making identification possible even if more difficult. However, there is one report in the literature of a non-secretory MTC, where none of these were present in detectable levels [9]. In these cases, it is suggested that pro-calcitonin or calcitonin gene related peptide (CGRP) may provide clues to diagnosis as they serve as alternative forms of calcitonin that may be produced by these altered C cells.

## 2 Case/case series presentation

A man, in his 30s, with past medical history of hypertension, hypothyroidism, and obesity was evaluated prior to enrollment in a clinical trial for a novel GLP-1 agonist for weight loss treatment. As part of the trial evaluation, serum calcitonin levels were drawn. Interestingly, the screening calcitonin was elevated to 115 pg/mL, with a repeat level of 131 pg/mL (normal range: 0–8.4 pg/mL). Therefore, he was deemed ineligible to join the clinical trial, and withdrew from the trial prior to further treatment. Upon follow up with his primary care doctor (PCP), who practices in an independent provider network outside of our hospital system, he was also noted to be clinically hypothyroid. This diagnosis was based on patient complaints of tiredness, weight gain, and constipation with a thyroid stimulating hormone (TSH) 6.5 uIU/mL (0.5–5.0 uIU/mL). Related labs include free T4 1.2 ng/dL (0.8–2.2 ng/dL) and free T3 5.3 pg/mL (2.8–5.3 pg/mL). He was started on levothyroxine supplementation by his PCP. On our exam, he endorsed symptoms of neck fullness, weight gain, fatigue, and altered stool frequency. He denied symptoms of headaches, palpitations, or sweating. General examination, including thorough skin exam was unremarkable. He was normotensive (BP 136/64) with a heart rate of 77. Upon our laboratory examination his calcitonin level remained high at 93 pg/mL after initiation of treatment for his hypothyroidism which can also lead to increased calcitonin levels.

He was referred to a multi-disciplinary endocrine tumor clinic where extensive family history and review of systems was completed. The patient reported no family history of thyroid, parathyroid, or pancreas malignancies. He did have a family history of prostate cancer. Evaluation with thyroid ultrasound revealed a 7 × 5 × 8 mm solid, isoechoic mid right thyroid lobe nodule, a 6 × 7 × 7 mm solid and hypoechoic left mid thyroid lobe nodule with microcalcifications, and several slightly enlarged central and left lateral neck lymph nodes. Bedside fine needle aspiration (FNA) evaluation was completed of the abnormal appearing left lateral neck level III lymph nodes, resulting in benign aspirates. Neither thyroid nodule met strict ATA criteria for biopsy given their size [10].

Further laboratory evaluation revealed normal calcium, normal parathyroid hormone (PTH), elevated carcinoembryonic antigen (CEA) of 5.2 ng/mL (normal <3 ng/mL), slightly elevated plasma normetanephrines of 1.1 (<0.9 nMol/L) and undetectable plasma metanephrines (<0.20 nMol/L). 24-hour urine normetanephrines were 846 (<482 mcg/day) and metanephrines were 133 (<190 mcg/day). CT abdomen and pelvis with IV contrast was performed due to mild elevation in normetanephrines and did not reveal an adrenal or extra-adrenal mass consistent with a catecholamine-producing tumor.

Two weeks after being seen in clinic, total thyroidectomy was planned with bilateral central lymph node dissection. After left thyroid lobectomy with central neck dissection was completed, the surgery was aborted due to ongoing hypoxia after ruling out potentially reversible causes intraoperatively. Postoperative calcitonin was 42 pg/mL (normal range: 0–8.4 pg/mL) and CEA level was not measured due to planned reoperation. The mid lateral left thyroid contained a focal 0.8 cm medullary thyroid carcinoma, 0.2 cm from the posterior margin, 0.2 cm from the anterior margin, and 3.5 cm from the isthmus margin, with focal lymphovascular invasion staged as a T1aN1a lesion. Five of the six resected lymph nodes contained evidence of metastatic MTC. There was no noted C-cell hyperplasia and sample has a low mitotic index (<2 per 2 mm^2).

Post-operatively, he was seen by a pulmonologist who diagnosed obesity hypoventilation syndrome, which improved with bronchodilators. He was euthyroid and it is not believed that his previously diagnosed hypothyroidism significantly contributed to his poor ventilation during surgery. Six weeks later, he next underwent completion thyroidectomy with right central lymph node dissection with final pathology demonstrating a 0.8 cm focal carcinoma in the mid right medullary thyroid, 0.2 cm from the anterior and posterior surfaces, and 1.7 cm from the isthmus margin, with negative margins and without lymphovascular invasion. There was no noted C-cell hyperplasia or angioinvasion and mitotic index was low (<2per 2mm^2). Final postoperative calcitonin was 12 pg/mL (normal range: 0–8.4 pg/mL) and CEA level was 2.0 ng/mL (normal <3 ng/mL). He was discharged post-operatively on day zero from both operations. Neither c cell mapping and ki67 measurements were completed on either pathology sample as they were taken prior to publication of the most recent WHO guidelines. Samples are unable to be retroactively tested at this time.

## 3 Discussion

The case presented in this report is notable because the patient had an indolent MTC that was detected incidentally because of a workup before starting a novel GLP-1 agonist, often used for diabetes management and weight loss [11]. Increases in calcitonin and increased risk of C-cell hyperplasia or MTC have been associated with the administration of a long acting GLP-1 receptor agonist in rat models [12]. So far, there are no data suggesting the same correlations exist in human recipients, and GLP-1 agonist carcinogenesis is not believed to be due to the same pathway that typically causes human C-cell cancers, the *RET* proto-oncogene [13,14]. However, it has been suggested that while GLP-1 agonists are not thought to cause MTC, the effect of increased calcitonin may accelerate the growth of MTC and, because of this growth, increase detection of previously undiagnosed MTC [15]. In our patient he did not receive the GLP-1 antagonist due to his diagnosis, so it cannot be determined if the GLP-1 antagonist would have caused growth or progression of his cancer.

Upon drug approval, the FDA requires systematic monitoring of the incidence of malignancies related to medications, including MTC related to new GLP-1 receptor agonists [15]. Limitations to gathering data on this relationship include reporting bias, the low incidence of MTC in the general population, and the expected long latency period from exposure to development of MTC.

Although calcitonin screening is not required nor recommended in all individuals before starting a GLP-1 agonist outside of a clinical trial, in the case of this patient, it aided in early identification of MTC and familial MEN 2 [14]. It is estimated that patients with a calcitonin level greater than 100 pg/mL have a 90–100 % chance of having MTC, and patients with a calcitonin of 25–100 have a 25 % chance of having MTC [16]. Calcitonin is not a marker specific to MTC or thyroid disease. It can be elevated in a range of diseases like other cancers, renal failure, Zollinger-Ellison syndrome, and pernicious anemia [17].

To date, there have not been any cost-effectiveness clinical trials for calcitonin screening prior to initiation of GLP-1 agonists. However, there are several cost-benefit analysis studies in the United States, China, and Germany within the last 10 years in patients with thyroid nodules, although these are hypothetical or expert opinion studies [[18], [19], [20]]. In the latest study in the United States, calcitonin screening appears to be cost effective in comparison to fine needle aspiration biopsy for diagnosis of MTC [18]. Of note, these studies have not specifically been connected with GLP-1 agonist therapy. The theoretical risk of diagnosing or developing C-cell hyperplasia or MTC may warrant future investigation into the cost-effectiveness of screening patients with calcitonin prior to starting GLP-1 agonist treatment outside of clinical trials. Additionally, further clinical trails may be indicated to see if the use of GLP-1 antagonists contribute to increased calcitonin levels or growth of MTC in humans.

## 4 Conclusion

This case presents a unique experience of a patient with mild presentation of familial MEN 2 which was only recognized as an incidental finding of elevated calcitonin as part of a clinical trial. Because symptomatic onset was mild, the progression of the patient's MTC may have gone unnoticed for a significant time before being detected. The incidental detection of elevated calcitonin not only caught this patient's MTC before it spread diffusely, but also allowed genetic testing of family members. Multidisciplinary care is key to treatment of patients with these multiple endocrine neoplasia syndromes as many organ systems may be involved and genetic counseling is paramount.

## CRediT authorship contribution statement

**Katie Glasgow:** Writing – review & editing, Writing – original draft, Conceptualization. **Victoria Jiminez:** Writing – review & editing, Writing – original draft. **Natalie Garcia:** Writing – original draft, Conceptualization. **Andrea Gillis:** Writing – review & editing, Supervision, Conceptualization.

## Declaration of competing interest

The authors declare that they have no known competing financial interests or personal relationships that could have appeared to influence the work reported in this paper.

## References

[1] Al-Salameh A., Baudry C., Cohen R. (2018). Update on multiple endocrine neoplasia Type 1 and 2. Presse Med [Internet].

[2] Gertner M.E., Kebebew E. (2004). Multiple endocrine neoplasia type 2. Curr Treat Options Oncol [Internet].

[3] Melmed S., Polonsky K.S., Reed Larsen P., Kronenberg H.M. (2011). Williams Textbook of Endocrinology.

[4] Wells SA Jr, Asa S.L., Dralle H., Elisei R., Evans D.B., Gagel R.F. (2015). Revised American Thyroid Association guidelines for the management of medullary thyroid carcinoma: the American thyroid association guidelines task force on medullary thyroid carcinoma. Thyroid.

[5] Păun D.L., Poiană C., Petriş R. (Nov-Dec 2013). Multiple endocrine neoplasia type 2A: case report. Chirurgia.

[6] Ungureanu S., Şipitco N., Alexa Z., Gonţa V., Bujac M., Parnov M. (2020). MEN 2A syndrome - multiple endocrine neoplasia with autosomal dominant transmission. Int J Surg Case Rep [Internet].

[7] Yasir M., Mulji N.J., Kasi A. (2021). StatPearls [Internet].

[8] Gambardella C., Offi C., Patrone R., Clarizia G., Mauriello C., Tartaglia E. (2019). Calcitonin negative medullary thyroid carcinoma: a challenging diagnosis or a medical dilemma. BioMed Central [cited 2023 Aug 19]. Available from.

[9] Gambardella C., Offi C., Clarizia G., Romano R.M., Cozzolino I., Montella M. (2019). Medullary thyroid carcinoma with double negative calcitonin and CEA: a case report and update of Literature Review - BMC Endocrine Disorders [Internet]. BioMed Central.

[10] Haugen B.R., Alexander E.K., Bible K.C., Doherty G.M., Mandel S.J., Nikiforov Y.E. (2016). 2015 American Thyroid Association Management Guidelines for adult patients with thyroid nodules and differentiated thyroid cancer: the American Thyroid Association Guidelines Task Force on thyroid nodules and differentiated thyroid cancer. Thyroid.

[11] Nauck M.A., Quast D.R., Wefers J., Meier J.J. (2021). GLP-1 receptor agonists in the treatment of type 2 diabetes – state-of-the-art. Mol. Metabol..

[12] Rosol T.J. (2013). On-target effects of GLP-1 receptor agonists on thyroid C-cells in rats and mice. Toxicol. Pathol..

[13] Lunati M.E., Grancini V., Colombo C., Palmieri E., Resi V., Perrino M. (2016). Basal and stimulated calcitonin levels in patients with type 2 diabetes did not change during 1 year of Liraglutide Treatment. Metabolism.

[14] Madsen L.W., Knauf J.A., Gotfredsen C., Pilling A., Sjögren I., Andersen S. (2012). GLP-1 receptor agonists and the thyroid: C-cell effects in mice are mediated via the GLP-1 receptor and not associated with Ret Activation. Endocrinology.

[15] Nauck M.A., Friedrich N. (2013). Do GLP-1–based therapies increase cancer risk?. Diabetes Care.

[16] Toledo S.P.A., Lourenço Jr DM., Santos M.A., Tavares M.R., Toledo R.A., Correia-Deur J.E. (2009). Hypercalcitoninemia is not pathognomonic of medullary thyroid carcinoma. Clinics.

[17] Sizemore G.W., Go V.L., Kaplan E.L., Sanzenbacher L.J., Holtermuller K.H., Arnaud C.D. (1973). Relations of calcitonin and Gastrin in the zollinger-ellison syndrome and medullary carcinoma of the thyroid. N. Engl. J. Med..

[18] Al-Qurayshi Z., Kandil E., Randolph G.W. (2020). Cost-effectiveness of routine calcitonin screening and fine-needle aspiration biopsy in preoperative diagnosis of medullary thyroid cancer in the United States. Oral Oncol..

[19] Hao W.J., Zhang H., Yu Y. (2019 Jul). Clinical significance and cost-benefit analysis of serum calcitonin assay in diagnosis and treatment of medullary thyroid carcinoma. Zhonghua er bi yan hou tou jing wai ke za zhi.

[20] Frank-Raue K., Schott M., Raue F. (2018). Empfehlung zum calcitonin-screening bei struma nodosa. Dtsch. Med. Wochenschr..

